# Racial and Ethnic Differences in Diet Quality by Education Level: Diminished Returns

**DOI:** 10.5888/pcd23.260004

**Published:** 2026-09-03

**Authors:** Chelsea R. Singleton, Ana Peralta-Garcia, Danielle J. Gartner, Kaustubh V. Parab, Isa Adamu, Oluwafikayo S. Adeyemi-Benson, Caryn N. Bell

**Affiliations:** 1 Department of Social, Behavioral, and Population Sciences, Celia Scott Weatherhead School of Public Health & Tropical Medicine, Tulane University, New Orleans, Louisiana; 2 Department of Epidemiology and Biostatistics, Dornsife School of Public Health, Drexel University, Philadelphia, Pennsylvania; 3 Aurora St. Luke’s Medical Center, Milwaukee, Wisconsin; 4 Department of Kinesiology & Community Health, College of Applied Health Sciences, University of Illinois at Urbana-Champaign, Champaign, Illinois

## Abstract

Higher education is correlated with healthy eating, which prevents chronic disease. Recent evidence suggests that racial and ethnic minority populations with higher education levels consume fewer healthy foods than their non-Hispanic White counterparts. We analyzed data on 19,591 participants of the National Health and Nutrition Examination Survey (2011–2018). Linear regression models identified racial and ethnic differences in Healthy Eating Index (HEI)–2015 scores within categories of education level. Non-Hispanic Black adults with a bachelor’s degree or higher had lower HEI–2015 scores (β = −1.69; SE = 0.79; *P* = .03) compared with non-Hispanic White adults with the same education level. These findings suggest that non-Hispanic Black populations in the US may experience diminished returns on educational investment.

**Summary**

**What is already known on this topic?**

National health data indicate that higher education is associated with better diet and health. However, emerging evidence suggests that some racial and ethnic minority populations may not experience the same return on educational investment as White populations.

**What is added by this report?**

This study documented significant racial and ethnic differences in diet quality within educational strata. Non-Hispanic Black adults with a bachelor’s degree or higher had lower diet quality than non-Hispanic White adults with the same level of education.

**What are the implications for public health practice?**

The field of public health should document the structural factors that perpetuate racial and ethnic dietary disparities across the spectrum of education and economic position.

## Objective

Suboptimal diet quality is a risk factor for chronic disease (1). In the US, diet quality varies by race and ethnicity, with studies reporting that non-Hispanic Black adults have poorer diet quality than non-Hispanic White adults (2). Because these disparities often persist when accounting for socioeconomic status, scholars have stressed the importance of considering structural factors when studying dietary disparities (3).

Disparities in diet quality also exist by education level (2). Historically, higher education has been positively associated with better dietary behavior because education improves health literacy and access to higher paying jobs (4). Yet, recent evidence suggests that the return on educational investment may not be similar across racial and ethnic groups (4,5). For example, Assari and Lankarani found that race moderated the effect of higher education on fruit and vegetable consumption, with non-Hispanic Black adults experiencing a reduced beneficial effect compared with non-Hispanic White adults (5). The authors describe this phenomenon as *diminished returns*, which occurs when socioeconomic resources (eg, income, education) yield weaker health and well-being benefits for racial and ethnic minority populations than for non-Hispanic White populations (5,6).

To our knowledge, no study has explored whether diminished returns are present regarding overall diet quality. We therefore examined racial and ethnic differences in diet quality by education level in a large sample of US adults.

## Methods

We analyzed cross-sectional data from 4 cycles (2011–2018) of the National Health and Nutrition Examination Survey (NHANES). In all cycles, NHANES oversampled Hispanic or Latino individuals and non-Hispanic Black individuals (7). In cycles 2013–2018, NHANES oversampled non-Hispanic Asian individuals (7). We analyzed data for adult participants aged 20 years or older (N = 22,617). After excluding participants without 24-hour recall data for day 1, the analytical sample comprised 19,591 adults (86.6%).

We modeled diet quality as measured by the Healthy Eating Index (HEI)–2015 as our dependent variable. HEI–2015 total score represents how well an individual’s food and beverage intake aligns with the 2015–2020 Dietary Guidelines for Americans (8). We scored each participant’s 24-hour recall data for day 1 using the simple scoring algorithm developed by the National Cancer Institute and the US Department of Agriculture (8). HEI–2015 scores can range from 0 to 100. A higher score indicates better diet quality.

Race and ethnicity (non-Hispanic Asian, non-Hispanic Black, Hispanic or Latino, non-Hispanic White, other race or multiracial) and education level (less than high school diploma, high school diploma or equivalent, some college or associate’s degree, or bachelor’s degree or higher) served as the primary independent variables. Covariates included age (years), sex (male or female), marital status (married, living with partner, or other), poverty level as a percentage of the federal poverty threshold (<100%, 100%–199%, 200%–299%, or ≥300%), number of household members, number of prepared meals purchased in the prior 7 days, and day of the week for the 24-hour recall interview (weekend vs weekday) (9).

We used SAS 9.4 (SAS Institute Inc) to conduct our analyses and accounted for the complex NHANES sampling scheme. We calculated weighted means and percentages stratified by education level to identify demographic differences across the education categories. Multivariable-adjusted linear regression models tested for racial and ethnic differences in HEI–2015 scores within the education categories. All models were adjusted for covariates, and α was set at .05. The data used for this secondary analysis are deidentified and publicly available. The institutional review board at Tulane University deemed this research exempt.

## Results

Of the total participants (N = 19,591), 4,171 (13.8%) participants had less than a high school diploma, 4,403 (22.9%) had a high school diploma or equivalent, 6,088 (32.5%) had some college or associate’s degree, and 4,929 (30.8%) had a bachelor’s degree or higher (Table 1). Compared with participants with a bachelor’s degree or higher, we found greater proportions of non-Hispanic Black participants, Hispanic or Latino participants, and participants living in poverty or near poverty among those with less than a high school education. HEI–2015 total scores, on average, were lower for lower education levels, including less than a high school diploma and high school diploma or equivalent (48.7 and 48.2, respectively), compared with higher education levels, including some college or an associate’s or bachelor’s degree or higher (49.9 and 55.7, respectively).

**Table 1:** Descriptive Characteristics of Adult Participants by Education Level, National Health and Nutrition Examination Survey, 2011–2018^a^

Variable	**All participants^b^**	Less than high school diploma	High school diploma or equivalent	Some college or associate’s degree	Bachelor’s degree or higher
**No. (%)**	19,591 (100.0)	4,171 (13.8)	4,403 (22.9)	6,088 (32.5)	4,929 (30.8)
**Age, mean (SD), y**	47.8 (17.6)	49.5 (17.3)	48.2 (18.1)	46.5 (17.9)	48.0 (16.6)
**Sex, no. (%)**					
Male	9,521 (48.1)	2,159 (51.9)	2,263 (51.0)	2,716 (44.6)	2,383 (47.9)
Female	10,070 (51.9)	2,012 (48.1)	2,140 (49.0)	3,372 (55.4)	2,546 (52.1)
**Race and ethnicity, no. (%)**					
Hispanic or Latino	4,652 (14.5)	1,978 (38.5)	975 (14.9)	1,146 (12.1)	553 (6.3)
Non-Hispanic Asian	2,281 (4.9)	278 (4.0)	293 (2.8)	439 (2.9)	1,271 (8.7)
Non-Hispanic White	7,480 (66.0)	992 (41.4)	1,792 (65.2)	2,595 (68.0)	2,101 (75.1)
Non-Hispanic Black	4,463 (11.2)	830 (13.4)	1,192 (13.6)	1,602 (12.6)	839 (7.1)
Other race and multiracial	715 (3.5)	93 (2.8)	151 (3.4)	306 (4.4)	165 (2.8)
**Marital status, no. (%)**					
Married or living with partner	11,527 (63.3)	2,457 (61.3)	2,434 (59.1)	3,355 (59.6)	3,281 (70.9)
Other	8,058 (36.7)	1,713 (38.7)	1,966 (40.9)	2,732 (40.4)	1,647 (29.1)
**Poverty level as percentage of federal poverty threshold, no. (%)**					
<100%	3,872 (14.5)	1,499 (36.1)	1,039 (18.7)	1,041 (13.7)	293 (3.6)
100%–199%	4,776 (20.6)	1,321 (34.4)	1,311 (27.1)	1,584 (22.5)	560 (8.5)
200%–299%	2,698 (15.3)	426 (13.9)	717 (20.1)	985 (17.6)	570 (10.2)
≥300%	6,487 (49.6)	388 (15.6)	949 (34.1)	2,008 (46.3)	3,142 (77.7)
**No. of household members, mean (SD)**	3.0 (1.7)	3.6 (1.9)	3.1 (1.7)	3.0 (1.6)	2.8 (1.4)
**No. of prepared meals in prior week, mean (SD)**	3.7 (3.9)	2.7 (3.4)	3.4 (3.7)	4.0 (4.0)	4.2 (4.0)
**Healthy Eating Index–2015 total score,^c^ mean (SD)**	51.1 (14.0)	48.7 (13.7)	48.2 (13.2)	49.9 (13.6)	55.7 (14.3)

a National Health and Nutrition Examination Survey sample weights were applied to calculate weighted means and percentages.

b Sample sizes may not sum to total sample size due to missing data.

c HEI–2015 scores can range from 0 to 100; higher scores indicate better diet quality (8).

Non-Hispanic Asian participants had significantly higher HEI–2015 scores than non-Hispanic White adults at all education levels (Table 2). Hispanic or Latino participants had significantly higher HEI–2015 scores than non-Hispanic White participants for all education levels except bachelor’s degree or higher, where we found no difference. HEI–2015 scores of non-Hispanic Black participants were significantly different from scores of non-Hispanic White participants among participants with a high school diploma or equivalent and a bachelor’s degree or higher. Among those with a high school diploma or equivalent, non-Hispanic Black participants had higher HEI–2015 scores than non-Hispanic White participants (β = 1.52; SE = 0.69; *P* = .03). However, non-Hispanic Black participants with a bachelor’s degree or higher had significantly lower HEI–2015 scores than non-Hispanic White participants (β = −1.69; SE = 0.79; *P* = .03) with the same education level. We observed no difference in HEI–2015 scores between non-Hispanic White participants and participants who self-reported their race and ethnicity as other race or multiracial.

**Table 2:** Stratified Linear Regression Models Examining Associations Between Race and Ethnicity and Healthy Eating Index–2015 Total Score, National Health and Nutrition Examination Survey, 2011–2018^a^

Race and ethnicity	β (SE) [*P* value]^b^				
	All participants (N = 19,591)	Less than high school diploma (n = 4,171)	High school diploma or equivalent (n = 4,403)	Some college or associate’s degree (n = 6,088)	Bachelor’s degree or higher (n = 4,929)
Hispanic or Latino	3.16 (0.52) [<.001]	4.96 (0.78) [<.001]	4.41 (0.92) [<.001]	1.52 (0.71) [.04]	0.20 (1.13) [.86]
Non-Hispanic Asian	6.04 (0.54) [<.001]	7.90 (1.33) [<.001]	8.20 (1.03) [<.001]	5.73 (0.93) [<.001]	4.13 (0.72) [<.001]
Non-Hispanic Black	−0.29 (0.39) [.47]	0.45 (0.69) [.52]	1.52 (0.69) [.03]	−0.53 (0.59) [.38]	−1.69 (0.79) [.03]
Non-Hispanic White	Reference	Reference	Reference	Reference	Reference
Other race and multiracial	0.58 (0.94) [.54]	−1.33 (1.89) [.48]	0.62 (1.08) [.57]	0.93 (1.07) [.39]	1.44 (2.25) [.52]

a National Health and Nutrition Examination Survey sample weights were applied to calculate weighted parameter estimates and SEs.

b Regression models adjusted for age, sex, marital status, poverty level as a percentage of the federal poverty threshold, number of household members, number of prepared meals, and day of the week of 24-hour recall interview (weekend vs weekday).

## Discussion

We examined racial and ethnic differences in diet quality by education level and found that non-Hispanic Asian adults had better diet quality than non-Hispanic White adults in all educational strata. Hispanic or Latino adults had better diet quality than non-Hispanic White adults, but only among those who had less than a high school education. Studies have reported that non-Hispanic Asian and Hispanic or Latino adults have better diet quality than non-Hispanic White adults (2). The composition of Asian and Latin diets, which often feature nutrient-dense foods consistent with high diet quality (eg, fruit, vegetables, whole grains) (10) may explain the difference in diet quality. The similarity in diet quality observed between Hispanic or Latino and non-Hispanic White adults in the higher education strata may be explained by dietary acculturation or increased access to financial resources (11–13). Dietary acculturation (adopting the food patterns of a culture after migrating) is associated with declines in diet quality among Hispanic or Latino populations (11,12). Moreover, people with more financial resources eat out more, which can expose them to ultraprocessed and calorically dense foods (10). Gaps in knowledge of the interplay between education and diet among Asian and Hispanic or Latino populations warrant more research.

We observed that among those with a bachelor’s degree or higher, non-Hispanic Black adults had poorer diet quality than non-Hispanic White adults, which may be evidence of diminished returns (6). Structural factors may explain why studies continue to report that Black adults with higher education and income have poorer health behaviors and outcomes compared with their White counterparts (4–6). The legacy of structural racism in America has reduced access to healthy food in Black communities (14). Black people, even at higher income levels, are more likely to live in communities where retailers selling unhealthy foods (eg, dollar stores, fast food restaurants) are more abundant (15). Furthermore, the Black–White wealth gap grew between 2019 and 2022 despite increases in educational attainment among Black adults (16). Our findings provide initial evidence of diminished returns on education level as it relates to diet quality. More research is needed to better describe the structural drivers of this phenomenon.

This study has several limitations. First, the cross-sectional design did not allow causal relationships to be established. Second, the data were self-reported and may have been affected by recall or reporting errors. Third, we analyzed data from NHANES cycles 2011–2018. Because HEI–2015 was developed after cycles 2011–2012 and 2013–2014, dietary data from those cycles are not aligned with the 2015–2020 Dietary Guidelines for Americans. Finally, we calculated HEI–2015 total score using the simple scoring algorithm and 1 day of 24-hour recall data. Because we did not use Markov Chain Monte Carlo methods for computing HEI (8), our results do not provide insight into usual dietary intake in this analytical sample.

We found racial and ethnic differences in diet quality by education level among US adults. These findings contribute to the growing science on drivers of racial and ethnic disparities in diet. Non-Hispanic Black adults may experience diminished returns on educational investment, with major implications for their dietary intake. Additional research is needed to identify the structural factors that contribute to the dietary disparity seen between non-Hispanic Black adults and non-Hispanic White adults.
